# Molecular identification of *Giardia* and *Cryptosporidium* from dogs and cats

**DOI:** 10.1051/parasite/2013008

**Published:** 2013-03-12

**Authors:** Isaia Sotiriadou, Nikola Pantchev, Doreen Gassmann, Panagiotis Karanis

**Affiliations:** 1 Medical and Molecular Parasitology Laboratory, Medical School, Centre of Anatomy, Institute II, University of Cologne Joseph-Stelzmann Str. 9 D-50931 Cologne Germany; 2 Centre of Dental Medicine, Department of Operative Dentistry and Periodontology, University of Cologne Kerpenerstr. 32 D-50931 Cologne Germany; 3 Idexx Vet Med Lab Moerikestrasse 28/3 D-71636 Ludwigsburg Germany

**Keywords:** *Cryptosporidium parvum*, *Giardia*, Assemblages, genotypes, zoonosis, Germany

## Abstract

The aim of the present study was to diagnose the presence of *Giardia* cysts and *Cryptosporidium* oocysts in household animals using nested polymerase chain reaction (PCR) and sequence analysis. One hundred faecal samples obtained from 81 dogs and 19 cats were investigated. The *Cryptosporidium* genotypes were determined by sequencing a fragment of the small subunit (SSU) *r*RNA gene, while the *Giardia* Assemblages were determined through analysis of the glutamate dehydrogenase (GDH) locus. Isolates from five dogs and two cats were positive by PCR for the presence of *Giardia*, and their sequences matched the zoonotic Assemblage A of *Giardia*. *Cryptosporidium* spp. isolated from one dog and one cat were both found to be *C. parvum*. One dog isolate harboured a mixed infection of *C. parvum* and *Giardia* Assemblage A. These findings support the growing evidence that household animals are potential reservoirs of the zoonotic pathogens *Giardia* spp. and *Cryptosporidium* spp. for infections in humans.

## Abstract (fr)

Le but de cette étude était de diagnostiquer la présence de kystes de *Giardia* et d’oocystes de *Cryptosporidium* chez des animaux de compagnie en utilisant la réaction en chaîne de la polymérase (PCR) et l’analyse des séquences. Cent échantillons de fèces obtenus de 81 chiens et 19 chats ont été étudiés. Les génotypes de *Cryptosporidium* ont été déterminés en séquençant un fragment de la petite unité (SSU) du gène *r*RNA, et les Assemblages de *Giardia* ont été déterminés par une analyse du locus de la glutamate déshydrogénase (GDH). Des isolats de cinq chiens et de deux chats ont été positifs par PCR pour la présence de *Giardia*, et leurs séquences correspondaient à l’Assemblage A de *Giardia*. Les *Cryptosporidium* isolées d’un chien et d’un chat appartenaient tous deux à l’espèce *C. parvum*. Un chien avait une infection mixte par *C. parvum* et *Giardia* Assemblage A. Ces résultats confirment que les animaux de compagnie sont des réservoirs potentiels des pathogènes zoonotiques *Giardia* spp. et *Cryptosporidium* pour des infections humaines.

## Introduction

*Cryptosporidium* and *Giardia* are protozoan pathogens that cause diarrhoeal illness when they colonise and reproduce in the intestines of humans or domestic animals, particularly dogs, cats, and livestock respectively. Both *Giardia* and *Cryptosporidium* are capable of completing their life cycle within a single host, resulting in cyst or oocyst stages that are excreted in the faeces. Epidemiological studies have focused on the transmission route of *Giardia* and *Cryptosporidium* [39, 40] and have sought to determine their zoonotic potential [45, 50]. *Giardia intestinalis* assemblages have been defined (A-H) by DNA sequence analysis so far, of which assemblages A and B are mainly virulent for humans [38, 49] and are often referred to as “zoonotic” assemblages [12, 27], but are also found in a number of other mammalian hosts [43]. Animals kept as household and/or pets play a significant role in the zoonotic transmission routes of the parasites due to their close association with their owners and the abundance of parasite cysts/oocysts excreted in large quantities [9, 16]. Household animals may also come into contact with free-living and/or domestic animals and can contract infections from them [9]. Animals can harbour infections of either zoonotic or host-specific *Giardia* Assemblages [14, 47, 48]. In general, cats and dogs may be affected by the host-specific *C. felis* and *C*. *canis,* respectively, and dogs can be infected with *C. parvum* due to the broader host range of this species [30]. Genotyping of oocysts recovered from the faeces of infected cats has shown that cats can also be infected with *C. muris* [36, 42]. *Giardia* host-adapted Assemblages in cats are usually A and F, whereas for dogs, the Assemblages include A, C and D [4, 21, 43].

The objective of the present study was to examine household animals for *Cryptosporidium* and *Giardia* infections.

## Materials and methods

### Sample collection

Faecal samples from domestic dogs and cats with clinical suspicion for giardiasis or cryptosporidiosis (predominantly diarrhoea of variable duration) were submitted by veterinary clinics from Germany and other European countries to a private veterinary laboratory in Germany (Idexx Vet Med Lab, Ludwigsburg, Germany). Fresh faecal samples from 81 dogs and 19 cats were collected during 2007 and were labelled and stored immediately at −20 °C. The samples were shipped to the laboratory at Cologne University for purification and processing and were kept frozen until used.

### Sample purification

Purification of the faecal samples and isolation of the oocysts/cysts were performed using the diethyl ether sedimentation technique combined with the saturated salt flotation technique, as described by Joachim et al. [22]. Briefly, each faeces sample was suspended in a 50 mL polypropylene tube with phosphate-buffered saline (PBS, pH 4) and homogenised by shaking vigorously. The excess faecal debris and lipids were removed by mixing the suspension with a one-fourth volume of diethyl ether until the sample became a homogenised emulsion; the suspension was then centrifuged at 2,500 *g* for 10 min. The supernatant was discarded and the remaining sediment was resuspended in distilled water and centrifuged twice as described above to remove other residues. The resulting pellet was resuspended in 5M cold saturated sodium chloride solution and carefully overlaid with cold distilled water so that a visible gradient was obtained. The samples were centrifuged at 2,300 g for 10 min, and oocysts/cysts were recovered from the interphase were washed twice with distilled water and stored in PBS (pH 7.4) with streptomycin (200 μg/mL) and amphotericin B (5 μg/mL) at 4 °C.

### DNA extraction

DNA was extracted from the purified faecal suspension and used for molecular analysis and sequencing. DNA extraction was performed using the modified method described by Karanis et al. [25] followed by the use of the QIAamp Stool Kit (Qiagen GmbH, Hilden, Germany). In brief, the oocysts/cysts were ruptured using 10 freeze-thaw cycles in the presence of lysis buffer in a dry thermo device (DTU-2B, Taitec, Japan) and were further processed according to the Qiagen manufacturer’s instructions. DNA was eluted in 100 μL AE buffer and stored at –20 °C.

### Molecular assays for the detection of *Giardia* and *Cryptosporidium* in faecal samples

To characterise the *Giardia* and *Cryptosporidium* species isolated from each positive sample, the DNA of all samples was extracted from the oocysts/cysts and was consequently tested by nested polymerase chain reaction (PCR). Positive faecal specimens were sequenced to identify the *Cryptosporidium* species and the *Giardia* Assemblages of the parasites found in the positive samples of the infected animals.

***Giardia* spp.** A fragment of the glutamate dehydrogenase (GDH) gene of approximately 220 bp in length was amplified using previously published primers and conditions [1]. All PCR amplifications were performed in an ABI 2720 Thermal cycler (Applied Biosystems, Foster, CA) in standard mixtures of 50 μL containing 1× PCR buffer, 200 nmol of each primer, 200 μM of dNTPs, 1.5 mM of MgCl_2_, 2.5 U of HotStarTaq DNA polymerase (Qiagen GmbH, Hilden, Germany), 2 μL of bovine serum albumin (BSA, acetylated, 10 mg/mL) (Promega, Madison, WI), 2 μL DNA template and distilled water. The PCR program included one incubation at 96 °C for 15 min and 40 amplification cycles (94 °C for 30 s, 55 °C for 30 s and 72 °C for 60 s), followed by one final extension incubation of 7 min at 72 °C. The PCR products were separated on a 1.6% agarose gel, stained with ethidium bromide and visualised on a UV transilluminator. PCR negative-control samples omitted template DNA, which was replaced by distilled water, and PCR positive-control samples, containing DNA extracted from 10 *Giardia* cysts, were always included for each test.

***Cryptosporidium* spp.** *Cryptosporidium* DNA was amplified by nested PCR using primers and conditions as described by Nichols et al. [32] and Karanis et al. [25] to produce a DNA fragment of approximately 440 bp in length of the SSU *r*RNA gene. PCRs were performed in 50 μL volumes containing 200 nmole of each primer, 1× PCR buffer, 200 μM dNTP, 3 mM MgCl_2_, 2.5 U GoTaq DNA polymerase (Promega, Wisconsin, USA), 2 μL BSA (10 mg/mL), 2 μL DNA template and distilled water. PCR consisted of one initial denaturation cycle at 96 °C for 15 min and 35 cycles (94 °C for 30 s, primary PCR 68 °C and secondary PCR 60 °C for 60 s, 72 °C for 30 s), followed by one final extension at 72 °C for 10 min. The PCR products were analysed as described above. Positive controls containing DNA extracted from 10 *C. parvum* oocysts and negative controls containing distilled water were included for each test.

### PCR product purification and sequencing

**Figure 1.**
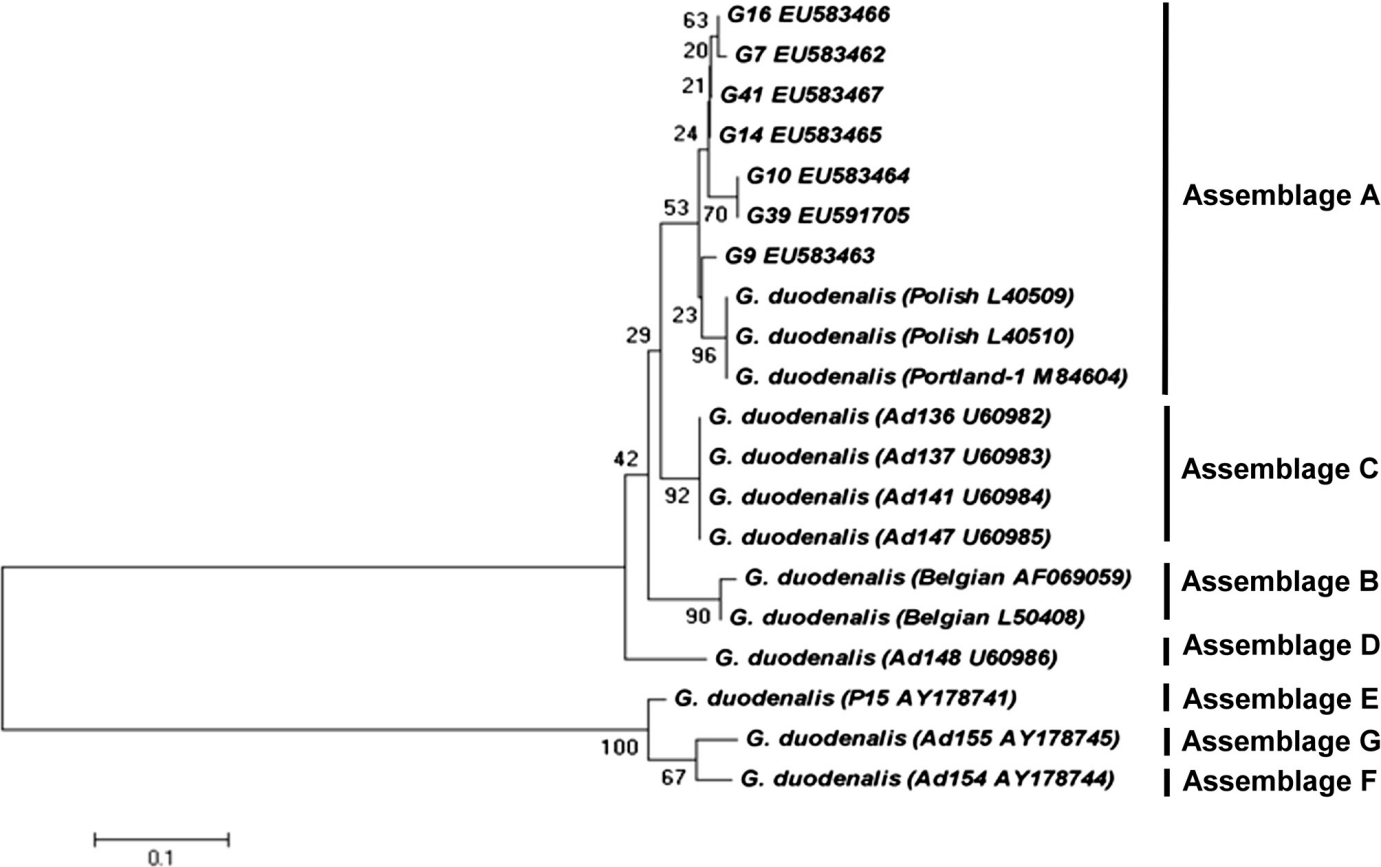
Phylogenetic relationship between the 7 *Giardia* Assemblages (G7, G9, G10, G14, G16, G41 and G39) obtained in this study and other previously published *Giardia* Assemblages. Nucleotide sequences of the GDH gene obtained in this study were aligned against reference sequences retrieved from GenBank using ClustalW and MEGA 5. The evolutionary history was inferred using the Neighbor-Joining method and the percentage of replicate trees in which the associated taxa clustered together in the bootstrap test (1,000 replicates) is shown next to the branches. The tree is drawn to scale, with branch lengths in the same units as those of the evolutionary distances used to infer the phylogenetic tree. The evolutionary distances were computed using the Kimura two-parameter method with the pairwise deletion option and are in the units of the number of base substitutions per site.

All PCR products from the molecular assays for both *Giardia* cysts and *Cryptosporidium* oocysts were cut out of agarose gels and purified with QIAquick Gel Extraction Kit (Qiagen GmbH, Hilden, Germany) according to the manufacturer’s instructions. The purified PCR products were directly sequenced in both directions on an ABI Prism 3100 (Applied Biosystems, Japan) Genetic Analyser using a Big Dye Terminator V.3.1 cycle sequencing kit (Applied Biosystems, Japan). The accuracy of the data was confirmed by two directional sequencing of the obtained sequences and by the alignment of the nucleotide sequences of the GDH gene for *Giardia* cysts and the SSU *r*RNA for *Cryptosporidium* oocysts against reference sequences retrieved from GenBank using the program ClustalW. Sequence similarity was also determined using the Basic Local Alignment Search Tool (BLAST) and the phylogenetic and molecular evolutionary analyses were conducted using MEGA version 5. The evolutionary history was inferred using the Neighbor-Joining method [41] and the respective evolutionary analyses were conducted using MEGA 5 [44]. The percentage of replicate trees in which the associated taxa clustered together in the bootstrap test (1,000 replicates) is shown next to the branches in Figure 1 [17]. The tree is drawn to scale, with branch lengths in the same units as those of the evolutionary distances used to infer the phylogenetic tree. The evolutionary distances were computed using the Kimura 2-parameter method [26] and are in the units of the number of base substitutions per site.

The nucleotide sequences of the positive samples have been submitted to the GenBank and they are available in the GenBank database under the Accession Nos. EU583462–EU583467 and EU591705 (93%–97% homology) for *Giardia* and EU606193 (94% homology) and EU606194 (99% homology) for *Cryptosporidium*.

## Results and Discussion

A total of 100 faecal samples from household animals, 19 cats and 81 dogs, were investigated by nested PCR for the presence of *Cryptosporidium* spp. oocysts and *Giardia* spp. cysts. To test for *Giardia,* all samples were amplified with the primers GDF3 and GDB5 and the PCR products were sequenced. Five dog and two cat faecal samples were found to be positive for *Giardia*. The results are presented in Table 1. In all cases, the samples found positive by PCR were confirmed by sequence analysis and no sequences were identified other than those of the typical *Giardia* Assemblage A in the dog and cat samples. In faecal samples of two dogs (6 year old, mix-breed with diarrhoea and vomiting and 1 year old with persistent diarrhoea), additional laboratory examinations identified the presence of *Escherichia coli* and *Campylobacter jejuni* (diagnosed by aerobe cultivation), respectively, but no other infections with yeasts and parasites have been found.

**Table 1.** Positive household animals for *Giardia* infections by PCR of the GDH locus, sequencing analysis results and accession numbers (*n* = 19 cats and *n* = 81 dogs).

Sample name	Species	*Giardia* PCR result of the investigation of the GDH locus	*Giardia* Assemblage by sequencing analysis	GenBank Accession No.
G7	Dog	+	A	EU583462
G9§	Cat	+	A	EU583463
G10	Dog	+	A	EU583464
G14	Dog	+	A	EU583465
G16	Cat	+	A	EU583466
G41	Dog	+	A	EU583467
G39*	Dog	+	A	EU591705

+ Positive.

§ Cat from Denmark.

* Dog with mixed infection (*Giardia* and *Cryptosporidium*).

SSU *r*DNA PCR and sequencing detected *Cryptosporidium* in one dog and one cat sample. Interestingly, both samples had high identity values to *C. parvum* (Table 2). Direct sequencing of these PCR products for both *Giardia* and *Cryptosporidium* showed that one dog carried a mixed infection with both *C. parvum* and *Giardia* Assemblage A (Tables 1 and 2). According to the study of Nichols et al. [33] outer amplification primers developed by them (N18SF2/R2) showed nonspecific amplification of DNA from the dinoflagellate *Gymnodinium*, but in both positive cases of our study, the results of nested PCR were confirmed by sequence analysis and no sequences were identified other than those of the typical *C. parvum*.

**Table 2.** Positive household animals for the presence of *Cryptosporidium* genotypes by PCR of the SSU *r*RNA and sequencing analysis results (*n* = 19 cats and *n* = 81 dogs).

Sample name	Species	*Cryptosporidium* PCR result of the investigation of the SSU *r*RNA gene	*Cryptosporidium* genotype by sequencing analysis	GenBank Accession No.
G39*	Dog	+	*C. parvum*	EU606193
G37	Cat	+	*C. parvum*	EU606194

+ Positive.

* Dog with mixed infection (*Giardia* and *Cryptosporidium*).

The phylogenetic relation of *Giardia* species is shown in Figure 1, using sequences of 13 *Giardia* Assemblages downloaded from GenBank as reference sequences. The phylogenetic tree indicates that the sequences obtained in our study cluster to *Giardia* Assemblage A. Even though the number of positive samples was relatively low, these results clearly demonstrate the presence of *Giardia* and *Cryptosporidium* among household animals from different regions in Germany.

According to a 5-year survey (1993–1997) of dairy herds in five German state veterinary laboratories, *Cryptosporidium* was diagnosed in 19–34% of the examined faecal samples and 20–36% of the post mortem cases [23] by conventional techniques. The primary cause of diarrhoea was acknowledged to be *Cryptosporidium* by only 1/5 of the investigators, indicating that the role of *Cryptosporidium* in Germany was underestimated. The recorded mixed infections were mostly associated with the presence of rotavirus and *E. coli*.

Only few studies have been carried out to evaluate the occurrence of different *Giardia* genotypes and *Cryptosporidium* species/genotypes in household animals in Germany and the actual risk of transmission to their owners. Broglia [11] genotyped *Cryptosporidium* isolates and defined the subtypes of *C. parvum* that originated from neonatal calves in Germany. All of the calf isolates in their study were identified as *C. parvum*. Other reports have also indicated that *C. parvum* is the most frequently found species of *Cryptosporidium* in pre-weaned calves [2, 37]. *Erinaceus europaeus* L. (European hedgehogs) in Germany were found to be naturally infected with *Cryptosporidium* in 29.8% by coproantigen analysis, while molecular analysis revealed IIa, IIc and VIIa subtype families of *C. parvum* [15].

The retrospective study of Barutzki and Schaper [5] reported a prevalence rate of 16.6% and 12.6% for *Giardia* infections in dogs and cats, respectively, in Germany between 1999 and 2002. The same authors reported that *Giardia* spp. were the most commonly found parasites using coprological examinations (coproantigen ELISA or SAF technique) with prevalence rates of 18.6% and 12.6% in dogs and cats, respectively [6].

*Giardia* human isolates belong to Assemblages A and B [13, 24]. Each Assemblage consists of two distinct subgroups; Assemblage A can be divided into A-I and A-II and Assemblage B can be divided into subgroups B-III and B-IV [3, 20]. Zoonotic genotypes of *Giardia* Assemblage A that are specific for hosts other than humans and Assemblages C and D were specifically assigned for dogs [21, 31, 38, 46]. In contrast, Assemblages A and B are not human-specific and infect a wider host range including dogs, cats, livestock and wildlife and are considered to have zoonotic potential. Barutzki et al. [7] investigated two different groups of dogs: one group presented clinical symptoms of gastrointestinal disorders and the other group was randomly selected. They concluded that 7% of the randomly selected *Giardia* positive dogs carried zoonotic species of *Giardia* belonging to Assemblage A. Interestingly, Leonhard et al. [29] examined asymptomatic dogs in southern Germany that were kept isolated or in groups and found that Assemblage A was most prevalent. They also identified Assemblages A and C, but Assemblages C and D were very rarely found.

Only one case was recorded as an outbreak of gastroenteritis caused by *C. parvum* after diagnosis by serological tests, such as ELISA for specific IgG, analysis of stool specimens, genotyping of the isolates and epidemiological analysis in Germany. In an August 2001 field training session, half of the recruits (*n* = 201) of the German armed forces became ill with acute gastroenteritis [10]. The zoonotic transmission of the disease was excluded after analysis of faecal droppings of the sheep that grazed in the area. Although the investigators were not able to identify the source of infection, the analysis of the risk factors correlated between drinking of tap water during the field exercise or the consumption of various meals at the beginning of the field training and gastroenteritis. Gallas-Lindemann et al. [18] examined a large area of 650 km^2^ in Lower Rhine, Germany, and provided substantial evidences on the dissemination and circulation of infected with (oo)cysts waste water. Deposition of (oo)cysts in waste water from households and their presence in all investigated water sources demonstrated the risk of waterborne transmission threatening for human health (from waste water through surface and groundwater to drinking water), since both parasites were detected in the whole surface water system [18].

*Giardia* and *Cryptosporidium* infections in household and domestic animals have been described also in other European countries highlighting the role of animals, and in particular of cats and dogs, in transmission of the infection to humans [8, 19, 28, 34, 35].

In spite of the small number of the indicated positive samples, the present study enhances the knowledge of the occurrence of *Giardia* spp. and *Cryptosporidium* spp. in privately owned household animals and genetically characterises the isolates found in the infected animals.

In summary, both *Giardia* and *Cryptosporidium* parasites are capable of infecting a variety of vertebrate species, from humans to animals, demonstrate a worldwide distribution, and are pathogens of veterinary and public health concern because of their ability to cause gastrointestinal disease, their ubiquitous presence in the environment, and the propensity for waterborne and foodborne outbreaks of these parasites. The absence of clinical symptoms results in the true prevalence of these parasites being underestimated, not only in cats and dogs but also in humans, and should be of major concern to veterinarians and physicians alike. Factors that may be affecting the underestimation of the risk could be related to poor and outdated information, improper interpretation of the published data and lack of sensitive methods. Infected household animals may especially pose a risk to immunocompromised people because they can be more susceptible to infection with *Giardia* and *Cryptosporidium.*
